# The Role of Matrix Metalloproteinase-9 in Atherosclerotic Plaque Instability

**DOI:** 10.1155/2020/3872367

**Published:** 2020-10-06

**Authors:** Tiewei Li, Xiaojuan Li, Yichuan Feng, Geng Dong, Yuewu Wang, Junmei Yang

**Affiliations:** ^1^Zhengzhou Key Laboratory of Children's Infection and Immunity, Children's Hospital Affiliated to Zhengzhou University, Henan Children's Hospital, Zhengzhou Children's Hospital, 33 Longhu Waihuan East Street, Jinshui District, Zhengzhou 450000, China; ^2^Children's Hospital Affiliated to Zhengzhou University, Henan Children's Hospital, Zhengzhou Children's Hospital, 33 Longhu Waihuan East Street, Jinshui District, Zhengzhou 450000, China; ^3^The Engineering Research Center for New Drug Screening, Inner Mongolia Medical University, Hohhot, China

## Abstract

Matrix metalloproteinase-9 (MMP-9) belongs to the MMP family and has been widely investigated. Excessive MMP-9 expression can enhance extracellular matrix degradation and promote plaque instability. Studies have demonstrated that MMP-9 levels are higher in vulnerable plaques than in stable plaques. Additionally, several human studies have demonstrated that MMP-9 may be a predictor of atherosclerotic plaque instability and a risk factor for future adverse cardiovascular and cerebrovascular events. MMP-9 deficiency or blocking MMP-9 expression can inhibit plaque inflammation and prevent atherosclerotic plaque instability. All of these results suggest that MMP-9 may be a useful predictive biomarker for vulnerable atherosclerotic plaques, as well as a therapeutic target for preventing atherosclerotic plaque instability. In this review, we describe the structure, function, and regulation of MMP-9. We also discuss the role of MMP-9 in predicting and preventing atherosclerotic plaque instability.

## 1. Introduction

Vulnerable plaques are a leading cause of acute coronary syndrome and sudden death. Studies have shown that 75% of acute coronary syndrome cases are caused by plaque rupture [1, 2]. According to the consensus document published in 2003 by Naghavi et al. [3], vulnerable plaques are defined as any types of plaques with a high likelihood of thrombotic complications and rapid progression and are characterized by a thin fibrous cap (<65 *μ*m), large lipid pool (>40% of the plaque), infiltration of inflammatory cells (particularly macrophages), outward remodeling, neovascularization, and intraplaque hemorrhage [4, 5]. Mature plaques mainly comprise endothelial cells (ECs), vascular smooth muscle cells (VSMC), macrophages, and a fibrous cap containing extracellular matrix (ECM) components [6]. Among these components, the ECM plays an especially important role in plaque stability [7].

Matrix metalloproteinases (MMPs) are members of the metzincin protease superfamily of zinc-endopeptidases. At present, the MMP family itself includes 28 members [8]. MMPs have a specific proteolytic activity against the ECM; their action can result in the thinning of the fibrous cap and plaque instability [6, 9]. MMP-9, also known gelatinase B, is a widely investigated member of the MMP family. Studies have shown that there is a strong relationship between MMP-9 and plaque instability [10–12]. Histopathological studies showed that MMP-9 was mostly distributed in the shoulder regions, necrotic core, and the fibrous cap of the atherosclerotic plaques, and that MMP-9 levels and activity were higher in unstable plaques than in stable plaques [13–16]. In addition, many studies have suggested that high MMP-9 levels can serve as a predictor of atherosclerotic plaque instability, and furthermore, that excessive MMP-9 expression may contribute to the plaque instability [17–19], indicating that MMP-9 may be a potential target for preventing atherosclerotic plaque instability. This review will explore the function of MMP-9 in vulnerable plaques and primarily discuss the predictive and preventive value of MMP-9 in atherosclerotic plaque instability.

## 2. Molecular Architecture and MMP-9 Regulation

### 2.1. MMP-9 Molecular Architecture

The majority of MMPs (with the except of matrilysins and MMP-26) have a similar structure, including a signal peptide, a propeptide, an active site, a Zn^2+^ binding domain, and a hemopexin domain [20, 21]. In addition, MMP-9 has an O-glycosylated domain and three fibronectin repeats (MMP-2 also has fibronectin repeats) [22, 23]. These structural domains form an inactive 92 kDa pro-MMP-9 or an active 82 kDa MMP-9 [24]. MMP-9 also exists in a third form as a 65 kDa protein that lacks the carboxyterminal hemopexin domain and amino terminal propeptide [25]. The catalytic domain of MMP-9 is composed of the active site and the zinc-binding sites and can be inhibited by propeptide. The propeptide region contains the “cysteine switch” consensus sequences PRCXXPD [26] and is degraded during enzyme activation. The native ECM is cleaved by collagenases MMP-1, MMP-3, or MMP-13 into 1/4 and 3/4 fragments, which are subsequently degraded further by other MMPs, such as MMP-2, MMP-3, and MMP-9 [27].

The sequence of the MMPs hemopexin domain is similar to that of plasma hemopexin, but MMP-9 only has 25%-33% sequence similarity with other MMPs [28]. The hemopexin domain of pro-MMP-9 binds to the C-terminal domain of tissue inhibitors of metalloproteinases (TIMPs) forming a tight complex [29]. MMP-9 can also bind to surface receptors that interact with substrates and induce autoactivation [24]. A number of studies have shown that the O-glycosylated (OG) domain, a unique linker sequence located between the hemopexin domain and the active site, is essential for MMP-9 function [20, 22, 30]. A deficiency in the OG domain significantly attenuates MMP-9-mediated cell migration and decreases gelatinolytic activity [31]. The OG domain also enables the catalytic domain and the terminal hemopexin domain of MMP-9 to move flexibly, which is important for denatured collagens degradation [24, 32].

MMP-9 exists in a variety of forms, including monomers, oligomers, a truncated form, or in a complex with other molecules [24, 33], and the monomer is the dominant form in human tissue and blood. The MMP-9 monomer can also form oligomers through intermolecular cysteine bridges. Dufour et al. [28, 30] reported that MMP-9 multimers can bind to CD44, further promoting the migration of cancer cells via activation of EGFR and the MAPK signaling pathway. Moreover, pro-MMP-9 trimers have a higher affinity for TIMP-1 than the monomers [34]. MMP-9 can also form a complex with neutrophil gelatinase-associated lipocalin (NGAL). The NGAL/MMP-9 complex, mainly secreted by neutrophils, is more stable than MMP-9 and can prevent MMP-9 from being degraded [35]. In addition, Winberg et al. [36] reported that macrophages secrete pro-MMP-9 covalently linked to the core protein of chondroitin sulfate proteoglycans via one or more disulphide bridges.

### 2.2. Regulation of MMP-9 Expression and Activity

#### 2.2.1. Regulation of MMP-9 Expression

The promoter region of the MMP-9 gene has many transcription factor binding sites, such as nuclear factor *κ*B (NF-*κ*B), activator protein-1 (AP-1), and specificity protein 1 (Sp-1) [37]. These transcription factors can enhance the transcription activity of MMP-9. MMP-9 can be secreted by multiple cell types, including macrophages, SMCs, and ECs. Bansal et al. [38] reported that macrophages expressed MMP-9 after stimulation with phosphatidyl-myo-inositol dimannosides (PIM2) through PI3K and Notch1 signaling pathways. SMCs also exhibited augmented expression of MMP-9, increased MMP-9 enzymatic activity, and impaired function of collagen assembly upon cross-talk with macrophages in high glucose conditions [39]. In addition, Magid et al. [40] found that the expression of MMP-9 in ECs is flow-sensitive and is upregulated by oscillatory flow via c-Myc activation. All of these effects on the regulation of MMP-9 expression may contribute to the development and progression of atherosclerosis.

#### 2.2.2. Activation of the MMP-9

Most MMPs are expressed and secreted as an inactive zymogens form, which is called pro-MMPs [41]. Activation of membrane-type MMPs (MT-MMPs) involves removal of the prodomain by furins in the endosomal pathway, while other MMPs rely on the cysteine switch mechanism [32]. MMP propeptide holds a cysteine which interacts with the active site zinc ion and thereby keeps the enzyme inactive. The cysteine switch mechanism means that pro-MMP activation requires disruption of the interaction between the cysteine switch and the zinc-binding site in the catalytic domain, either by proteolytic removal of the propeptide or by chemical modification of the cysteine. As reviewed by Yabluchanskiy et al. [37], MMP-9 can be activated by multiple MMPs and proteolytic enzymes, such as MMP-2, MMP-3, MMP-13, MMP-17, MMP-26, and proteolytic enzymes such as urokinase-type plasminogen activator (uPA), plasmin, and tissue-type plasminogen activator. In addition, low pH, sodium dodecyl sulfate (SDS), other denaturing agents, and heat treatment can also activate MMP-9 by cleaving its predomain [42].

#### 2.2.3. Endogenous Inhibitors of MMPs

TIMPs are the main physiological inhibitors of MMPs in vivo. Of the four known TIMPs, TIMP-1 inhibits MMP-9 with high-affinity [43, 44]. The C-terminal domain of TIMP-1 can bind with the hemopexin domain of pro-MMP-9 to form a tight complex, thus inhibiting MMP-9 activation, as well as TIMP-3 [29]. Several studies have shown that MMP-9 and TIMP-1 are coexpressed in many types of cells (except neutrophils that do not express TIMP-1) and are secreted as an MMP-9/TIMP-1 complex [30, 45]. However, Serifova et al. [46] found that homotrimeric MMP-9 could efficiently cleave the alpha-2-macroglobulin (*α*2M), induce *α*2M conformational changes (*α*2M∗), and form covalent complexes (*α*2M∗/MMP-9), and hence escape protease inhibition and internalization. Endogenous inhibitors and MMP-9 are in a dynamic balance under physiological conditions, but this balance will be disrupted under certain pathological conditions.

## 3. MMP-9 and Atherosclerosis

Acute coronary events, approximately 75% of which are caused by unstable plaque rupture, present a serious threat to human life [1, 2]. The transition from stable to unstable plaques involves ECM remodeling, and altered MMP-9 activity accelerates unstable plaque development [47–49].

### 3.1. Role of MMP-9 in Atherosclerotic Plaque Instability

The presence of MMP-9 protein in human coronary atherosclerotic lesions was first reported by Brown et al. in 1997 [50]. Many subsequent studies have shown that MMP-9 is involved in the process of atherosclerosis development [51, 52]. When under stress, ECs exhibited augmented MMP-9 expression and activity [40]. Increased MMP-9 expression and activity promote ECM degradation, which can enhance inflammatory cell infiltration. Histopathological studies showed that MMP-9 was mostly distributed in the shoulder regions, necrotic core, and the fibrous cap of carotid atherosclerotic plaques [53] that contained an abundance of inflammatory cells [54]. The inflammatory cells are the primary source of MMP-9 within the plaque [55]. Chen et al. [56] reported that macrophage-derived MMP-9 could promote the infiltration of monocyte/macrophages into the lesions but had no effect on the fatty streak size. Only overexpression of the autoactivating form of MMP-9 in macrophages can induce significant plaque disruption [57]. In addition, MMP-9 can also promote the migration of VSMCs. VSMC can further secrete vascular endothelial growth factor (VEGF), which plays an important role in neovascularization (a risk factor for plaque instability). Papalambros et al. [58] reported that MMP-9 and VEGF are expressed in parallel in neovascularized plaque lesions. MMP-9 may influence the stability of plaques indirectly via effects on neovascularization. Animal experiments have shown that plaque volume and length are significantly reduced in MMP-9-deficient mice. Carotid plaques in these mice accumulated less SMC, collagen, intraplaque foam cells, and macrophages [59]. MMP-9 deficiency also impairs compensatory vessel enlargement [47]. However, MMP-9 has a dual role in atherosclerosis, as reviewed by Newby et al. [55]. MMP-9 could facilitate the contact between SMCs and the interstitial matrix through degrading the ECM around VSMCs, resulting in a change from quiescent, contractile VSMCs to cells capable of migrating, proliferating, and subsequent intimal thickening. Lemaître et al. [60] reported that overexpression of pro-MMP-9 in macrophages did not induce plaque rupture but resulted in augmented collagen deposition, suggesting that MP-9 had a protective role in plaque in instability. Johnson et al. [61] further demonstrated that loss of MMP-9 significantly reduced SMC content, increased plaque area, and resulted in higher macrophage infiltration. The extent to which these two opposite effects on stability occur in vivo remains to be elucidated.

**Figure 1:**
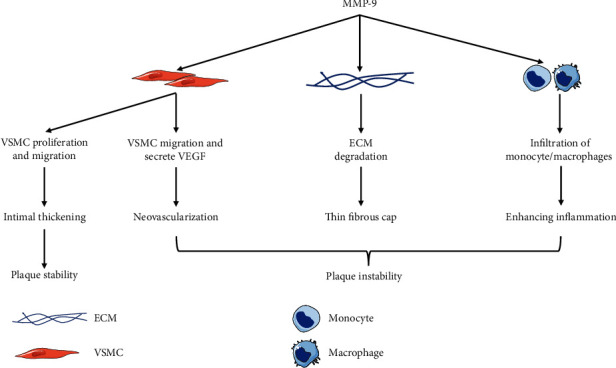
Emerging roles of MMP-9 in atherosclerosis, atherothrombosis, and vulnerable plaques. Abbreviations: MMP-9: matrix metalloproteinase-9; ECM: extracellular matrix; VSMC: vascular smooth muscle cell; VEGF: vascular endothelial growth factor.

MMP-9 is also present in atherosclerotic lesions of coronary arteries and the aorta. Jiang et al. [62] found that MMP-9 expression was elevated in porcine coronary artery intima with unstable plaques. Morishige et al. [63] further reported that overexpression of MMP-9 in porcine coronary arteries after balloon injury promoted thrombus formation, and that MMP-9 deficiency reduced the aorta's atherosclerotic burden and suppressed macrophage infiltration, while loss of MMP-12 did not affect the lesion growth. This difference in effect may be due to the difference in substrates between MMPs; MMP-9 disrupts the basement membrane collagen, initiating the development of atherosclerosis, while MMP-12 mainly degrades the elastic laminae of atherosclerotic media without affecting plaque growth [64]. Loss of MMP-9 can also prevent aortic dilatation and the formation of abdominal aortic aneurysms (AAA) [65]. Silencing of MMP-9 can also reduce the C-reactive protein levels (CRP) in aortic atherosclerotic plaques indicating that MMP-9 deficiency may stabilize the plaques by inhibiting their inflammation [66]. Galis et al. [67] further found that targeted disruption of the MMP-9 gene can prevent SMC migration and limit the effects of pathological arterial remodeling in restenosis and atherosclerosis. More importantly, a clinical study reported that the expression of the MMP-9 gene and protein is more abundant in unstable plaques, and that transcription factors AP-1 and NF-*κ*B are more frequently detected in unstable plaques that have been shown to upregulate MMP-9 expression [11]. A synopsis of potential MMP-9-mediated mechanisms driving plaque instability has been visualized in Figure 1.

### 3.2. Predictive Value of MMP-9 in Atherosclerosis

At a diagnostic or prognostic level, circulating MMP-9 levels have been suggested to be a biomarker for predicting the stability of atherosclerotic plaques and the risk of future adverse cardiovascular and cerebrovascular events. Fukuda et al. [19] reported that the serum levels of MMP-9 were elevated in patients with ruptured plaques compared with the patients without ruptured plaques, and that MMP-9 was an independent predictor of plaque rupture. Elevated serum levels of MMP-9 also have a positive association with high total carotid artery plaque score, larger intima-media thickness (IMT) value, and plaque instability [68]. Olson et al. [69], however, did not find an association between plasma MMP-9 concentration and carotid plaques, but total and active MMP-9 concentration were associated with femoral artery plaques and IMT. These differences between studies may be due to the different types of samples evaluated in each study. Several studies have shown that MMP-9 levels are higher in serum than in plasma and found a stronger association between MMP-9 levels and neutrophils in serum than in plasma, possibly due to leucocyte activation during the clotting process [69, 70]. MMP-9 levels also vary between patients with acute myocardial infarction (AMI) and unstable angina pectoris (UAP); serum MMP-9 levels are higher in patients with ST segment elevation myocardial infarction (STEMI) than in those with non-STEMI (NSTEMI) or UAP [71]. There is a higher frequency of plaque rupture, thin-cap fibro atheroma (TCFA), and red thrombi in patients with STEMI than in those with NSTEMI, suggesting that MMP-9 may be released from vulnerable plaques [72]. However, elevated levels of MMP-9 in plaque tissue may not result in a higher level of circulating MMP-9 in the blood [73]. In addition, active MMP-9 was efficiently complexed to *α*2M in human plasma, resulting in a shift of active MMP-9 into high molecular weight complexes, which could not be detected by standard analysis methods [46]. Therefore, the detrimental MMP-9 activity may persist due to the *α*2M∗/MMP-9 trimer complexes.

MMP-9 can be secreted by many types of inflammatory cells, including monocytes, macrophages, neutrophils, and foam cells [24]. Under normal physiological conditions, peripheral blood monocytes express low levels of MMP-9, but MMP-9 expression increases under pathological conditions when it is upregulated by many proinflammatory factors and the ECM [74–76]. Brunner et al. [77] reported that the monocytic MMP-9 mRNA expression is higher in patients with UAP/NSTEMI than in healthy individuals or patients with stable angina pectoris (SAP). Many studies have reported that MMP-9 levels are strongly associated with neutrophils, where MMP-9 is abundant in neutrophils [78, 79]. Compared with the de novo production of MMP-9 by macrophages, neutrophils have MMP-9 prestored in secretory granules for quick release under stimuli [80]. These findings suggest that MMP-9 may be secreted by inflammatory cells. In addition, plasma MMP-9 levels are significantly increased in infarct-related arteries than in the femoral artery and peripheral vein and are positively correlated with the volume of myocardial infarction (MI) area, suggesting that MMP-9 may also be released from the infarcted myocardium [81–83].

The expression of MMP-9 also has temporal and spatial features. MMP-9 levels are significantly increased in patients with MI and return to baseline within one week [82]. Inokubo et al. [84] reported that the plasma MMP-9 levels are significantly increased in coronary circulation, but not in the aortic root. Circulating MMP-9 levels are further elevated after mechanical disruption of plaques by percutaneous coronary intervention [85]. These findings suggest that the elevation of MMP-9 in coronary circulation may be due to secretion by vulnerable plaques. Another study reported that the MMP-9 levels significantly increased more in early acute coronary syndrome (ACS) (≤4 h after oneset) than in late ACS (>4 h after oneset). In contrast, high-sensitivity troponin T (hs-TnT) levels are lower in early ACS than in late ACS [71]. This study also reported that MMP-9 plasma levels increased earlier than levels of hs-TnT. MMP-9 is thus a better marker than hs-TnT for the diagnosis of early ACS, as it reflects plaque rupture or vulnerability [71].

Although many studies have shown that MMP-9 can serve as a biomarker of vulnerable plaques, its predictive value for adverse cardiovascular and cerebrovascular outcomes is controversial. Hamed et al. [86] reported that patients with ACS who have adverse cardiovascular events have a higher level of MMP-9. MMP-9 levels can also be used to predict ischemic stroke and cardiovascular death in patients with > or =50% carotid stenosis [87]. However, Jefferis et al. [88] pointed out that serum MMP-9 is not a strong predictor for MI and stroke in older men and women. The possible explanation for these inconsistent results may be that the two studies have different populations. In the general population, serum MMP-9 levels are associated with the incidence of coronary heart disease (CHD) [89], but the odds ratio decreased significantly to borderline after adjustment for conventional risk factors (especially smoking) [90]. In a study by Eldrup et al. [91], MMP-9 levels were not a valuable predictor for UAP, MI, or death in patients with stable coronary disease, while CRP levels could predict poor disease outcomes. The controversy over the predictive value of MMP-9 for adverse cardiovascular outcomes requires further evaluation in future studies. Eighteen studies of the association between circulating MMP-9 levels and atherosclerosis are summarized in Table 1.

### 3.3. MMP-9 Gene Polymorphisms in Atherosclerosis

The human MMP-9 gene is located on chromosome 20q12.2-13.1. A number of single nucleotide polymorphisms (SNPs) are present in the promoter, coding, and untranslated regions of the MMP-9 gene, the 1562C>T polymorphism in the promoter and the 279 polymorphism (R279Q) in the coding region being of special interest. The 1562C>T polymorphism in the MMP-9 gene results in a cytosine base replacing a thymine base at position -1562 [92]. The C allele has higher affinity for binding the transcriptional repressor protein than the T allele, resulting in enhanced MMP-9 promoter activity. The R279Q polymorphism of the MMP-9 gene refers to the replacement of amino acid arginine (R) with glutamine (Q) in the catalytic domain of MMP-9, enhancing its binding ability to substrates [93, 94]. Zhang et al. [95] reported that the 1562C>T polymorphism of the MMP-9 gene affects MMP-9 gene transcription, resulting in high (C/T, T/T) or low (C/C) promoter activity. Further analysis showed that there is an association between the 1562C>T polymorphism in the MMP-9 gene and CHD severity. In addition, Wu et al. [96] reported that the 1562C>T polymorphism in the MMP-9 gene may be a predictor of susceptibility to ischemic stroke. Patients with the T/T genotype tended to have larger areas of complicated and calcified lesions than patients with the C/C genotype, while the MMP-9 genotype did not have a significant association with the degree of coronary artery stenosis [97].

However, there are some differences between reports on the relationship between the 1562C>T polymorphism in the MMP-9 gene and cardiovascular disease (CVD). Morgan et al. [98] found that there was an association between the 1562C>T polymorphism of the MMP-9 gene and CVD in the Caucasian population, while the 1562C>T polymorphism was only associated with the early-onset coronary artery disease (age <55 years) in the Iranian population [99]. In the Chinese Uighur population, the 1562C>T polymorphism of the MMP-9 gene is also significantly associated with MI, with a synergistic effect being seen among smokers [100, 101]. Additionally, Wu et al. [102] reported that the 1562C>T polymorphism in the MMP-9 gene was associated with coronary artery disease (CAD) in the Chinese Han population. Some studies, however, have pointed out that there are no significant associations between the 1562C>T polymorphism in the MMP-9 gene and CVD in patients from Korean [103], Turkey [104], the Caucasus [105, 106], Germany [92], and Italy [107]. Several meta-analyses have been carried out to evaluate the association between the 1562C>T polymorphism of the MMP-9 gene and CVD, with conflicting results. Zhang et al. [108], in a meta-analysis of 26 studies, suggested that the 1562C>T polymorphism was not associated with CAD in the overall population. However, results from two smaller meta-analyses showed that patients with T alleles had a higher risk of MI than patients with the C/C genotype and found a significant association between the 1562C>T polymorphism and CHD, but not in the Asian population [109, 110]. Interestingly, studies have shown no association between the R279Q polymorphism in the MMP-9 gene and CAD [100, 101, 106], but the R279Q polymorphism and smoking had a synergistic effect and were significantly associated with the risk of MI in the Chinese Uighur population [100, 101]. Opstad et al. [106] have reported that the R279Q polymorphism also had a significant association with hypertension. These contradictory results may be due to the differences in the types of patients, ethnicities, and countries in the studies analyzed. Further large-scale studies are needed to verify these findings and provide personalization of medical services for patients based on their countries, ethnicities, MMP-9 gene polymorphisms, and comorbidity. Studies about the association between MMP-9 gene polymorphisms and atherosclerosis are described in Table 2.

## 4. Pharmacotherapy Targeting MMP-9 in Atherosclerosis

TIMPs, the endogenous inhibitors of MMPs, play an important role in inhibiting MMPs activity. To date, four TIMPs have been identified, all of which can inhibit MMPs, but with different affinities. TIMP-1 can inhibit most of the MMPs (MMP-1, MMP-3, and MMP-9 in particular). TIMP-2 binds with good affinity to MMP-2. TIMP-3 is a powerful inhibitor of MMPs that are present in the ECM. TIMP-4 exhibits tissue-specific expression and is especially expressed in the heart [44, 111]. De Vries et al. [112] reported that TIMP-1 overexpression significantly attenuated plaque progression and increased the stability of vulnerable plaques in murine vein grafts. Compared with TIMP-1, two studies found that TIMP-2 played a greater protective role than TIMP-1 in preventing atherosclerotic plaque development.

In addition to TIMPs, synthetic inhibitors against MMPs have also been shown to be effective, and of these, doxycycline has been approved by the American Food and Drug Administration for clinical practice [113, 114]. Doxycycline inhibits the expression and activity of MMP-9 [115, 116], and doxycycline treatment can significantly inhibit atherosclerosis development [117]. Doxycycline can also reduce the neointimal thickness and has a protective effect on left ventricular dilation [118]. However, a human study demonstrated that administration of doxycycline had no effect on MMP-9 expression in carotid plaques and plaque rupture [119]. Brown et al. [120] further demonstrated that although doxycycline induced a 50% reduction in plasma pro-MMP-9 activity and exerted potentially beneficial effects on inflammation, it had no effect on the composite endpoint of sudden death, fatal MI, nonfatal MI, or troponin-positive UAP.

In addition, ONO-4817 is an ora MMP inhibitor that shows a broad spectrum of inhibitory activity. In an animal study, ONO-4817 treatment significantly inhibited MMP-9 activity within one day after MI oneset and attenuated the left-ventricular (LV) dilatation, as well as the development of cardiac dysfunction [121]. In addition, treatment with the MMP inhibitor PD166793 could also significantly reduce the infarct area and LV dilatation two weeks after MI [122]. However, MMP inhibitor PG-116800 failed to demonstrate that it could prevent LV remodeling and reduce the incidence of adverse cardiovascular events in the Prevention of Myocardial Infarction Early Remodeling (PREMIER) trial [123]. In addition, Johnson et al. [124] reported that broad-spectrum MMP inhibitor RS-130830 did not have a beneficial effect on atherosclerosis in the apolipoprotein E knockout mouse model.

Many studies have shown that common cardiovascular agents can also inhibit the expression or activity of MMP-9. Statins, widely applied in the clinical setting for hyperlipemia treatment, can inhibit the atherosclerosis development, stabilize the vulnerable plaques, and further reduce the adverse cardiovascular events [125, 126]. Statins have also been shown to could restore the EC function, inhibit proliferation and migration of VSMCs, and attenuate inflammatory response [127]. Luan et al. [128] reported that statins could inhibit the MMP-9 secretion from rabbit SMCs and foam cells in a dose-dependent manner and had no effect on the production of TIMP-1, thereby disrupting the balance between MMP-9 and TIMP-1. Rival et al. [129] further demonstrated that MMP-9 secretion was inhibited by fluvastatin via the mevalonate starvation mechanism. In addition, a clinical study showed that the levels of MMP-9 significantly decreased in patients receiving statins [130]. Takai et al. [131] reported that lisinopril therapy, but not candesartan cilexetil, significantly inhibited both the activity of angiotensin-converting enzyme and MMP-9. Patients given imidapril had a lower MMP-9 activity than those given lisinopril, indicating that different hypertension drugs had different inhibitory effects on MMP-9 activity [132]. In addition, insulin treatment can also effectively reduce the diabetes-increased intimal lesion size and MMP-9 expression but has no effect on TIMP-1 expression and macrophage content of atherosclerotic plaques [133]. Several studies have also shown that the antidiabetic PPAR-activator rosiglitazone significantly reduces serum MMP-9 levels in type 2 diabetes mellitus patients [134]. A number of other compounds, such as estradiol [135, 136], lycopene [137], polyphenols [138], panax notoginsenosides (TPNS) [139], and tanshinone IIA [140] can also affect MMP-9 levels and activity.

## 5. Conclusions

Vulnerable plaques remain a high risk factor for cardiovascular and cerebrovascular diseases, as their rupture can cause acute coronary syndrome, stroke, and sudden death. Vulnerable plaques are characterized as those having a thin fibrous cap, a large lipid pool, and infiltration of inflammatory cells. Inflammatory cells can release large amounts of MMP-9 into plaque tissue, which can then degrade the ECM, resulting in a thin fibrous cap and plaque instability. Numerous studies have demonstrated that MMP-9 levels are higher in vulnerable plaques than in stable plaques, and high circulating levels of MMP-9 are closely related to adverse cardiovascular and cerebrovascular events [48, 53, 62, 141–143]. Depletion of the MMP-9 gene or inhibition of MMP-9 expression or activity can significantly prevent plaque instability. All of these results suggest that MMP-9 can be a predictor of atherosclerotic plaque instability, and that targeting MMP-9 can prevent atherosclerotic plaque instability. However, we need to consider the effect of some factors on the relationship between MMP-9 and plaque instability, such as countries, ethnicities, MMP-9 gene polymorphisms, and other underlying diseases. Further large-scale studies and meta-analyses are needed to evaluate the synergistic effects and provide personalization of medical services for people based on their countries, ethnicities, MMP-9 gene polymorphisms, and comorbidities. Although there have been some clinical studies on the clinical application of MMP-9 inhibitors, the study populations have been relatively small. In addition, specific inhibitors of MMP-9 are still lacking. Further studies should continue to develop specific MMP-9 inhibitors and evaluate their role in preventing plaque instability.

## Figures and Tables

**Table 1:** List of studies showing associations between circulating MMP-9 levels and atherosclerosis.

Author, reference	Study design	Patient populations	Main study findings
Fukuda et al. [19]	Cross-sectional study	47 AMI patients, 23 UAP patients, and 19 SAP patients	Patients with plaque rupture had significantly higher levels of MMP-9 than patients who did not have plaque rupture.
Tan et al. [68]	Human study	116 stroke-free participants	Elevated serum MMP-9 concentration was independently associated with high total carotid artery plaque score, plaque instability, and large IMT value.
Olson et al. [69]	Cross-sectional study	473 61-year-old men	Plasma MMP-9 concentration was higher in men with echolucent femoral plaques, while no similar associations were found for carotid plaques.
Kobayashi et al. [71]	Human study	200 patients with ST elevation ACS and 66 patients with non-ST elevation ACS	MMP-9 levels significantly increased in early ACS than late ACS, while the hs-TnT levels were lower in early ACS than late ACS. Meanwhile, MMP-9 levels were elevated earlier than hs-TnT and had a higher diagnostic value for early ACS.
Tziakas et al. [73]	Human study	18 carotid specimens and serum obtained from 18 patients	MMP-9 levels measured in extracts from the most stenotic area were significantly higher in patients with intraplaque hemorrhage. However, serum levels of MMP-9 showed no difference.
Brunner et al. [77]	Human study	18 SAP patients, 14 UAP/NSTEMI patients, 14 STEMI patients, and 16 healthy controls	Monocytic MMP-9 mRNA levels were increased in patients with UAP/NSTEMI or STEMI compared to the controls and patients with SAP.
Koizumi et al. [81] Kaden et al. [82] Funayama et al. [83]	Human study		Plasma MMP-9 levels were significantly increased in infarct-related artery than those in femoral artery. MMP-9 levels returned to baseline by 1 week after MI.
Inokubo et al. [84]	Human study	29 ACS patients, 9 UAP patients, 17 SAP patients, and 20 control subjects	Plasma MMP-9 levels were significantly increased in coronary circulation, but not in aortic root.
Higo et al. [85]	Human study	23 AMI patients and 10 SAP patients performing percutaneous coronary intervention	Plasma MMP-9 levels were significantly higher in patients with AMI and further increased after percutaneous coronary intervention.
Hamed et al. [86]	Human study	75 ACS patients, 25 SAP patients and 20 healthy participants	Patients with ACS having adverse cardiovascular events had higher levels of MMP-9.
Eldrup et al. [87]	Human study	Followed up 207 patients with > or =50% carotid stenosis initially for a mean of 4.4 years	Elevated MMP-9 was associated with the risk of stroke and cardiovascular death.
Jefferis et al. [88]	Prospective study	368 incident MI patients and 299 incident stroke patients and two controls per case. Follow up for 8 years	Serum MMP-9 was not a strong independent risk marker for MI and stroke.
Garvin et al. [89]	Human study	428 men and 438 women (45-69 years), free of previous coronary events and stroke. Follow up for 8 years	Plasma MMP-9 was independently associated with the risk of first-time CHD.
Welsh et al. [90]	Human study	Followed up 5661 men for 16 years	MMP-9 was unlikely to be a clinically useful biomarker for CHD after adjustment for conventional risk factors (especially smoking).
Eldrup et al. [91]	Human study	Followed up 1090 patients with stable coronary heart disease for 15years	Elevated matrix metalloproteinase-9 had no association with increased risk of unstable angina, MI and death in patients with stable coronary heart disease.

Abbreviations: MMP-9: matrix metalloproteinase-9; MI: myocardial infarction; AMI: acute myocardial infarction; UAP: unstable angina pectoris; SAP: stable angina pectoris; hs-TnT: high sensitivity troponin T; ACS: acute coronary syndrome; STEMI: ST-elevation myocardial infarction; NSTEMI: non-ST segment elevation myocardial infarction; CHD: coronary heart disease.

**Table 2:** List of studies showing relationship between MMP-9 gene polymorphisms and atherosclerosis.

Author reference	Study design	Patient populations	Polymorphic site	Main study finding
Zhang et al. [95]	Human study	584 male patients with MI and 645 age-matched male healthy controls	1562C>T	1562C>T polymorphism of MMP-9 gene affects MMP-9 gene promoter activity, and has an effect on atherosclerotic phenotype.
Wu et al. [96]	Meta-analysis	Enrolled 19 studies including 5630 ischemic stroke cases and 5368 controls	1562C>T	1562C>T polymorphism also significantly correlated with the risk of patients with large artery atherosclerosis. Meanwhile, it also significantly correlated with the risk of ischemic stroke.
Pollanen et al. [97]	Autopsy study	276 men	T/T and C/C genotype	Patients with T/T genotype of MMP-9 had a larger area of complicated and calcified lesion compared with the patients with the C/C genotype of MMP-9, while no significant association between the MMP9 genotype and severity of atherosclerosis was found.
Morgan et al. [98]	Human study	1510 white subjects	1562C>T	1562C>T polymorphism of MMP-9 gene had an effect on MMP-9 expression, which can influence the development and progression of atherosclerosis.
Saedi et al. [99]	Human study	53 early-onset coronary artery disease patients and unrelated late-onsets CAD patients.	1562C>T	1562C>T polymorphism of MMP-9 gene was only associated with the early-onset coronary artery disease (age <55 years).
Wang et al. [100]	Case-control study	384 Chinese Uighur patients with MI proven by coronary angiography and 451 sex-matched and ethnically matched control participants	1562C>T and R279Q	1562C>T polymorphism of MMP-9 gene was significantly associated with MI, while R279Q polymorphism was not significantly associated with MI in Chinese Uighur population. Meanwhile, the -1562C>T or R279Q polymorphism of the MMP-9 gene and smoking had a synergistic effect and were significantly associated with the risk of MI.
Wang et al. [101]	Case-control study	361 ACS Chinese Uighur patients and 432 control subjects	1562C>T and R279Q	1562C>T polymorphism of MMP-9 gene was associated with ACS susceptibility in the Chinese Uygur population. However, R279Q polymorphism of the MMP-9 gene was not significantly associated with the risk of ACS.
Wu et al. [102]	Case-control study	258 CAD patients and 153 controls from the Chinese Han population	1562C>T	They found that the 1562C>T polymorphism of the MMP-9 gene was associated with CAD.
Kim et al. [103] Alp et al. [104] Wang et al. [105] Opstad et al. [106] Blankenberg et al. [92] Nuzzo et al. [107]	Human study		1562C>T	No significant association between the 1562C>T polymorphism of MMP-9 gene and CVD.
Zhang et al. [108]	Meta-analysis	Enrolled 26 studies including 12,776 cases and 6371 controls	1562C>T	1562C>T polymorphism of MMP-9 gene was associated with the risk of CAD in Asian populations, not in overall population.
Wang et al. [109]	Meta-analysis	Enrolled 18 studies including 9569 MI cases and 7264 controls	1562C>T	1562C>T polymorphism of MMP-9 gene was a risk factor of MI, but these associations varied in different ethnic populations.
Juan et al. [110]	Meta-analysis	Enrolled 7 studies including 3952 MI cases and 4977 healthy controls	1562C>T	1562C>T polymorphism of MMP-9 gene was a risk factor of MI in the total population and white populations, but not in Asian populations.

Abbreviations: MMP-9: matrix metalloproteinase-9; MI: myocardial infarction; ACS: acute coronary syndrome; CAD: coronary artery disease; SNP: single nucleotide polymorphism; CVD: cardiovascular disease.
